# Proteome Profiling Reveals NQO2 Activity Contributing to Proteasome Inhibitor Resistance in Multiple Myeloma Cell Lines

**DOI:** 10.1016/j.mcpro.2026.101615

**Published:** 2026-06-26

**Authors:** Chien-Yun Lee, Seungbin Han, Larissa Haertle, Umair Munawar, Cornelia Vogt, Johanna Streubel, Andrej Besse, Lenka Besse, Christoph Driessen, Hermann Einsele, Johannes Waldschmidt, Martin Kortüm, Bernhard Kuster

**Affiliations:** 1 Chair of Proteomics and Bioanalytics, School of Life Sciences, Technical University of Munich, Freising, Germany; 2 Department of Internal Medicine II, University Hospital Würzburg, Würzburg, Germany; 3 Department of Medicine III, Technical University of Munich, School of Medicine and Health, TUM University Hospital, Munich, Germany; 4 Laboratory of Experimental Oncology, Division Oncology and Hematology, HOCH Health Ostschweiz, Cantonal Hospital St Gallen, St. Gallen, Switzerland; 5 Department of Biology, Faculty of Medicine, Masaryk University, Brno, Czech Republic; 6 Bavarian Biomolecular Mass Spectrometry Center (BayBioMS), Technical University of Munich, Freising, Germany

**Keywords:** multiple myeloma, proteasome inhibitor resistance, quantitative proteomics, phosphoproteomics, NQO1, NQO2, ABCB1

## Abstract

Proteasome inhibitors (PIs) are frontline therapies for multiple myeloma (MM). Although MM patients initially respond to PIs, resistance frequently emerges. While all PIs nominally target the same proteasomal catalytic subunit (PSMB5), the extent to which resistance mechanisms are the same or different among different PIs or between patients is poorly understood. To address this, we performed proteome and phosphoproteome profiling of 12 MM cell line models, comprising four parental lines (AMO-1, ARH77, L363, and RPMI8226) paired with lines that acquired resistance to bortezomib (BTZ) or carfilzomib (CFZ). Over 7000 proteins and up to 10,000 phosphopeptides were identified per cell line, enabling a comprehensive comparative analysis of shared and cell line-specific resistance signatures at the protein level. Data analysis revealed surprisingly few changes in the phosphoproteome but substantial reprogramming of the proteome in most models. Beyond known adaptations such as the overexpression of the PI target PSMB5 and the drug efflux transporter ABCB1, we identified the oxidoreductases NQO1 and NQO2 as significantly upregulated proteins under chronic proteotoxic stress across several models. Pharmacological follow up in PI resistant AMO-1 cells showed that NQO2 inhibition by imatinib fully restored CFZ sensitivity, validating NQO2 as a contributor to resistance formation in this model system.

## Highlights

• Deep proteome profiling of 12 Multiple Myeloma (MM) cell models of PI resistance.
• Massive proteome reprogramming observed in PI-resistant MM cell lines.
• Identification of high NQO2 activity as a contributor to acquired drug resistance.
• Inhibition of NQO2 activity by imatinib restores sensitivity to carfilzomib.

## Graphical Abstract

**Figure:**
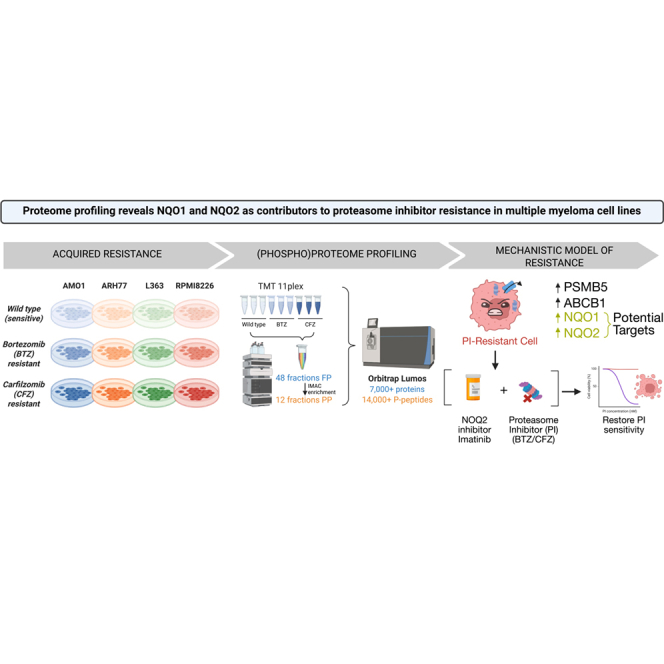

## In Brief

Proteasome inhibitor resistance in multiple myeloma was investigated through comprehensive proteome and phosphoproteome profiling of 12 cell line models, revealing substantial proteomic reprogramming and consistent upregulation of oxidoreductases NQO1 and NQO2. Pharmacological targeting of NQO2 with imatinib restored carfilzomib sensitivity, identifying a potential, actionable strategy to overcome drug resistance.

Multiple myeloma (MM) is a malignancy of terminally differentiated plasma cells that accumulate in the bone marrow, leading to cytopenia, renal dysfunction, and lytic bone disease (1, 2). Although the introduction of proteasome inhibitors (PIs) has significantly improved outcomes (3, 4), MM remains incurable due to the eventual development of drug resistance and most patients relapse with PI-refractory disease (5, 6). First-line regimens often include bortezomib (BTZ), a reversible boronate-based PI, or carfilzomib (CFZ), an irreversible inhibitor, both targeting the β5 catalytic subunit of the 20S proteasome core (PSMB5) (7, 8, 9). The efficacy of PIs stems from the high secretory burden of MM cells. Blocking the proteasomal degradation of misfolded or excess proteins (10, 11) induces proteotoxic stress and activates the unfolded protein response (UPR), ultimately triggering apoptosis (12, 13). The 20S proteasome comprises three catalytic subunits, β1, β2, and β5, with β5 being the primary therapeutic target (14). However, efficient cytotoxicity often requires co-inhibition of β2 or β1, especially in resistant contexts (15).

Resistance to PIs arises through diverse and complex mechanisms. These include (1) drug-induced overexpression of and mutations in PSMB5 that impair PI binding (16, 17, 18); (2) upregulation of ABCB1 (multi-drug resistance protein 1), promoting CFZ efflux and reducing intracellular drug levels (, 19, 20); (3) downregulation of the IRE1/XBP1 unfolded protein response (UPR) axis, reducing apoptotic signalling (21); and (4) broad metabolic rewiring, enhancing redox buffering and mitochondrial adaptation (19). Among these mechanisms, proteasome adaptation in myeloma cells has been widely discussed. Proteasome subunit alterations can influence PI sensitivity. While β5 is the primary drug target, functional studies have shown that myeloma cell viability is actually more dependent on the β1 and β2 proteolytic activities and that cells can better tolerate the partial loss of β5 activity (22, 23). This plasticity implies that even without PSMB5 mutations, resistant cells might survive by (i) relying on remaining activity of the other proteases in the complex subunits (including inducible immunoproteasome components) or (ii) by increasing bulk proteasome abundance (16, 24). Collectively, PI resistance in MM is a multifactorial phenomenon involving both genetic and adaptive changes that reduce drug-triggered proteotoxicity (25). Furthermore, while CFZ and BTZ both target β5, their differential susceptibility to drug efflux and proteasome subunit inhibition suggests distinct resistance mechanisms. For instance, CFZ's ability to inhibit β2 in addition to β5 is critical for overcoming resistance in certain models (26). Taken together, PI resistance is highly heterogeneous and may arise from convergent or clone-specific pathways (27), highlighting the need for broader comparative analyses.

While extensive genomic and transcriptomic studies have provided important insights into the mutational landscape, clonal evolution, and transcriptional rewiring associated with PI resistance in MM (28, 29, 30, 31), these molecular layers cannot capture the dynamic post-translational landscape that mediates the actual drug response. Proteomics and phosphoproteomics bridge this gap by directly measuring the functional effector proteins and phosphorylation-controlled signaling networks that govern cellular survival. Recently, advanced proteomic and multi-omic technologies have been successfully deployed to profile primary MM patient bone marrow samples, yielding insights into the broader proteogenomic landscape of the disease (32), tumor immune microenvironment dynamics (33), and produced novel prognostic biomarkers (34). However, while these approaches have powerfully illuminated general MM biology, the quantitative characterization of paired PI-sensitive and resistant MM cell lines at the proteome level has been less comprehensively studied (19, 35).

To identify additional resistance mechanisms at the protein level, we performed deep proteome and phosphoproteome profiling across 4 MM cell line models (parental AMO1, ARH77, L363, and RPMI8226) and their BTZ- and CFZ-adapted/resistant derivatives. By capturing approximately 7,000 proteins and 10,000 phosphopeptides per cell line, we mapped the molecular signatures contributing to PI resistance. While the phosphoproteome remained broadly stable, resistant lines recurringly exhibited significant upregulation of specific proteins. Among these were well-established players, notably the direct target of proteasome inhibitors PSMB5 and the efflux pump ABCB1. In addition, NQO1 and NQO2, both oxidoreductases involved in detoxification of xenobiotics and the regulation of redox homeostasis, were upregulated in many models. Functional validation confirmed that NQO2 overexpression can contribute to CFZ resistance, as inhibition of NQO2 activity by imatinib fully restored sensitivity to CFZ in AMO-1 cells. Collectively, our data provide a rich resource for the MM community for further research into PI resistance and establish the NQO enzyme family as potential therapeutically actionable mediators of PI-refractory disease.

## Experimental procedures

### Experimental Design and Statistical Rationale

To systematically investigate proteome and phosphoproteome adaptations driving proteasome inhibitor (PI) resistance, we employed a quantitative, multiplexed proteomics workflow. Four multiple myeloma (MM) cell line lineages (AMO-1, ARH77, L363, and RPMI8226) were utilized, comparing wild-type (WT) parental controls against their matched, *in vitro* drug-resistant (bortezomib, BTZ; carfilzomib, CFZ) derivatives. All cell culture, drug treatments, and omics preparations were performed in independent biological triplicates (same clone, different passages), yielding nine samples per lineage.

For LC-MS/MS analysis, the nine samples (biological triplicate of wild-type, BTZ- and CFZ-resistant lines) per lineage were digested and TMT 11-plex labelled. To complete the 11-plex design and enable cross-plex normalization, the remaining two channels in each batch were used for two identical aliquots of a pooled internal reference consisting of replicate one of the WT versions of each cell line. Samples were then fractionated for parallel global proteome and IMAC-enriched phosphoproteome profiling. Statistical significance of differential protein and phosphosite abundance was determined using two-sample independent Welch’s t-tests, followed by Benjamini-Hochberg false discovery rate (FDR) correction. Significant regulation was defined by a dual threshold of FDR <0.05 and an absolute fold-change ≥2. Inferred kinase activity was assessed via Student's *t* test (*p* < 0.05) requiring an absolute score difference >1. Downstream functional viability assays were also performed and evaluated in biological triplicates.

### Generation of Bortezomib- and Carfilzomib-Resistant Cell Lines

AMO-1, ARH77, L363, and RPMI8226 cell lines were cultured in RPMI-1640 medium (Gibco, ThermoFisher Scientific) supplemented with 10% FBS, 1% penicillin/streptomycin (Invitrogen), 1% GlutaMAX, and 1% sodium pyruvate (Gibco). Parental (wild-type) cells were authenticated at the Leibniz Institute DSMZ. Bortezomib (BTZ) and carfilzomib (CFZ) were obtained from Selleck Chemicals. Resistant cells were generated and characterized as described (19, 23) and maintained at a density of 0.4 to 1.2 × 10^6^ cells/ml in the presence of 90 nM of the respective PI, except for the RPMI-BTZ line, which was cultured with 60 nM BTZ. Drug solutions were freshly prepared from concentrated stocks. Cell cultures were maintained at 37°, 5% CO_2_ and routinely tested negative for *Mycoplasma*.

### Cell Viability Assay

Cell viability was assessed using the Alamar Blue assay. Cells were seeded in 96-well plates and treated with PIs (BTZ, CFZ), the NQO2 inhibitor imatinib, the ABCB1 inhibitor tariquidar either as single agents or in combination for 72 h. In drug combination experiments, imatinib (10 μM), and tariquidar (50 nM) were used at fixed concentrations while PIs were tested across a range of concentrations (1.25–1280 nM). After treatment, 20 μl of Alamar Blue reagent was added to each well and plates were incubated for 6 to 7 h at 37 °C. Absorbance was measured at 570/600 nm using a Tecan Infinite 200 pro plate reader. Cell viability measurements were normalized to drug vehicle (DMSO) controls. The half-maximal inhibitory concentration (IC_50_) was determined using a Python (v3.10) script by fitting mean viability data from biological replicates to a two-parameter logistic dose-response model (Hill equation): as follows

$$f\left(x\right)=\frac{100}{1+{\left(\frac{x}{{IC}_{50}}\right)}^{H}}$$

where f(x) is the percentage of cell viability, x the drug concentration, IC_50_ is the drug concentration required to inhibit viability by 50%, and H denotes the Hill slope (absolute value). To ensure numerical stability and facilitate logarithmic visualization, zero-dose control concentrations were adjusted to a baseline value of 0.01 nM. Dose–response curves are visualized on a symmetric logarithmic scale, with error bars indicating the standard error of the mean (SEM) across triplicates.

### Cell Lysis

Cells were washed twice with cold PBS and lysed in a buffer containing 40 mM Tris-HCl (pH 7.6) and 2% SDS. Cell lysates were boiled at 95 °C for 5 min, and trifluoroacetic acid (TFA) was added to a final concentration of 1% to reduce viscosity by hydrolysing DNA (36). To quench the acid, 3 M Tris (pH 10) was added to a final concentration of 195 mM, adjusting the pH to 7.8. Protein concentration was determined using the Pierce BCA Protein Assay Kit (Thermo Scientific). Biological triplicates from each cell line were prepared for subsequent analysis.

### Protein Digestion

Lysates were processed using the SP3 method on an automated Bravo liquid handling system (Agilent Technologies) as previously described (37) with minor modifications (38). Briefly, 20 μl of carboxylate bead mix (50 μg/μl in H_2_O; Sera-Mag SpeedBeads, cat# 45152105050250 and 65152105050250, GE Healthcare, Chicago, IL, USA) were added to a 96-well plate (cat# 951020401, Eppendorf, Hamburg, Germany). Following one precipitation step with 60% acetonitrile (CAN), two wash steps with 80% ethanol, and one wash step with 100% ACN, the beads were incubated with 100 μl of digestion buffer (50 mM HEPES, pH 8.5; 10 mM TCEP; 50 mM CAA) for 1 h at 1200 rpm and 37 °C for reduction and alkylation. Samples were subsequently digested overnight at 37 °C (1200 rpm) using a 1:50 trypsin-to-protein ratio.

The bead supernatant containing tryptic peptides was transferred to a new 96-well plate and acidified with formic acid (FA) to a final concentration of 1%. Peptides were desalted using RP-S cartridges (5 μl bed volume, Agilent) via the standard peptide cleanup v2.0 protocol on the AssayMAP Bravo Platform. Briefly, cartridges were primed with 100 μl of 50% ACN/0.1% FA and equilibrated with 50 μl of 0.1% FA (10 μl/min). Samples were loaded at 5 μl/min, followed by an internal wash with 0.1% FA (10 μl/min). Peptides were eluted with 80 μl of 70% ACN/0.1% FA (5 μl/min), dried down, and stored at −80 °C until further use.

### TMT Labelling and Multiplexing

Desalted peptides were labelled with TMT 11-plex reagents as previously described (39). Briefly, 100 μg of TMT reagent in 5 μl of anhydrous ACN was used to label 100 μg of peptides in 20 μl of 50 mM HEPES buffer (pH 8.5). The labeling reaction was stopped by adding 3 μl of 5% hydroxylamine. Samples from each channel were pooled in equal amounts and dried to remove the ACN. The pooled peptides were dissolved in 0.1% FA, desalted using 250 mg Sep-Pak C18 cartridges (Waters, Milford, MA, USA), and vacuum dried. In total, four batches of TMT10 plexed samples (lot numbers: TMT10plex, UF291262; TMT11, UE277617) were prepared (one batch per cell lineage). Each batch consisted of the nine experimental samples (triplicates of wild-type, BTZ-resistant, and CFZ-resistant cell lines) from the same parental cell line and two aliquots of a pooled sample (consisting of replicate one of the WT versions of each cell line) to act as bridge channels between TMT batches.

### Off-Line HPLC Fractionation

TMT-labelled peptides were fractionated by off-line basic reversed-phase (bRP) chromatography as previously described (40), with slight modifications. Briefly, a Dionex Ultra 3000 HPLC system equipped with an XBridge BEH130 C18 column (3.5 μm, 4.6 × 250 mm, Waters) was operated at a flow rate of 1 ml/min with a constant 10% of 25 mM ammonium bicarbonate (pH 8.0) in both solvents. A 57-min linear gradient from 7 to 45% ACN, followed by a 6-min linear gradient up to 80% ACN, was performed. Ninety-six fractions were collected every half minute from minute 7 to 55 and pooled into 48 fractions (fraction 1 + 49, fraction 2 + 50, etc.). To acidify the samples, 50% (v/v) FA in water was added to a final concentration of 1% (v/v). For global proteomics analysis, 75 μl (∼15% of the total peptide amount) was transferred to a new 96-well plate. The rest of the sample was kept for phosphopeptide enrichment. Both plates were dried down and stored at −80 °C.

### Phosphorylation Enrichment

The 48 bRP HPLC fractions were dissolved in IMAC equilibrium buffer (80% ACN/19.9% ddH_2_O/0.1% TFA) and pooled into 12 fractions following the same logic described above. Phosphopeptides from each fraction were enriched using Fe(III)-NTA cartridges (5 μl bed volume, Agilent) using the Phosphopeptide Enrichment v2.0 protocol on the AssayMAP Bravo Platform. Briefly, cartridges were primed with 150 μl of IMAC priming buffer (99.9% ACN/0.1% TFA) and equilibrated with 150 μl of IMAC equilibrium buffer at a flow rate of 10 μl/min. Next, samples were loaded at 5 μl/min, followed by three internal cartridge washes with IMAC equilibrium buffer at 50 μl/min. Phosphorylated peptides were eluted with 50 μl of 1% ammonia at a flow rate of 5 μl/min directly into 50 μl of 10% formic acid. Samples were dried down and stored at −80 °C until LC–MS/MS analysis.

### LC-MS/MS Analysis

#### Full Proteomes

For full proteome profiling, LC-MS/MS was performed using a Dionex UltiMate 3000 RSLCnano System equipped with a Vanquish pump module and coupled to a Fusion Lumos Tribrid mass spectrometer (Thermo Fisher Scientific) using micro-flow conditions as previously described (41). Peptide fractions were dissolved in 1% FA/2% ACN, and approximately 3 to 4 μg of peptides were injected directly onto an Acclaim PepMap 100 C18 column (2 μm particle size, 1 mm ID × 150 mm; Thermo Fisher Scientific). Peptides were separated at a flow rate of 50 μl/min using a 25-min linear gradient from 4 to 32% solvent B (0.1% FA and 3% DMSO in ACN) in solvent A (0.1% FA and 3% DMSO in water). Data-dependent acquisition (DDA) with an H-ESI source was utilized. MS1 full scans were recorded in the Orbitrap (OT) (360–1600 m/z, 60k resolution, AGC target 4e5, maximum injection time [maxIT] 50 ms). MS2 spectra were acquired in the ion trap (IT) (HCD 32% NCE, AGC 1.2e4, maxIT 40 ms, isolation window 0.6 m/z, intensity threshold 1e4). Quantitative information for TMT reporter ions was obtained by synchronous precursor selection (SPS) of the eight most intense fragments (38), followed by HCD (55% NCE) and MS3 acquisition in the Orbitrap (100–1000 m/z, 50k resolution, isolation window 1.2 m/z, AGC 1e5, maxIT 86 ms). The cycle time was 1.2 s with a 50 s dynamic exclusion.

#### Phosphoproteomes

For phosphoproteome profiling, peptides were dissolved in 0.1% FA, and half was loaded onto a trap column (75 μm × 2 cm, packed in-house with 5 μm C18 resin; Reprosil PUR AQ, Dr Maisch). After washing with 0.1% FA at 5 μl/min for 10 min, peptides were transferred to an analytical column (75 μm × 45 cm, packed in-house with 3 μm C18 resin; Reprosil PUR AQ, Dr Maisch). Peptides were separated at 300 nl/min using an 80-min linear gradient from 4 to 32% solvent B (0.1% FA and 5% DMSO in ACN) in solvent A (0.1% FA and 5% DMSO in water). DDA was performed using a high-resolution OT method. MS1 spectra were recorded in the OT (360–1500 m/z, 60k resolution, AGC 4e5, maxIT 50 ms). MS2 spectra were recorded in the OT (15k resolution, CID 35% CE, isolation window 0.7 m/z, neutral loss mass 97.9763). SPS-MS3 quantification of the 10 most intense fragment ions was performed via HCD (55% NCE) and recorded in the OT (100–1000 m/z, 50k resolution, isolation window 1.2 m/z, AGC 1.2e5, maxIT 120 ms). The cycle time was 3 s with a 90 s dynamic exclusion.

### Data Analysis

#### Raw Data Processing

The raw data files were searched using MaxQuant 1.6.2.10 against a human reference database provided by UniProt (downloaded 30 November 2020, containing 42,373 protein sequences including isoforms) and common contaminants. For full proteome analysis, the experiment type was set to 11plex TMT as an isobaric label within a reporter ion MS3 with a false discovery rate (FDR) cutoff of 1% at both the peptide and protein levels. Trypsin/P was selected as the proteolytic enzyme, allowing a maximum of two missed cleavages. Precursor mass tolerances were set to 20 ppm for the initial search and 4.5 ppm for the main search, while the fragment ion mass tolerance was set to 20 ppm. TMT 11-plex modifications on peptide N-termini and lysine residues, alongside cysteine carbamidomethylation, were considered as a fixed modification. Methionine oxidation and N-terminus acetylation were allowed as variable modifications. The default score cutoffs required a minimal Andromeda score of 40 and a delta score of six for modified peptides. Protein quantification was obtained from summed peptide reporter intensities. For phosphoproteomes, the settings mentioned above were used with modifications. STY phosphorylation was added to the variable modifications, and matching was enabled between fractions of samples in a 20-min alignment window and 0.7-min matching window.

#### Data Post-Processing

For the proteome, MaxQuant "proteinGroups.txt" files were utilized, while the phosphoproteome was analyzed from the "p10_evidence.txt" files generated by subjecting the initial search results to SIMSI-Transfer (v0.5.0) with a p10 threshold (42). Phosphorylation sites were annotated using a Python package (https://github.com/kusterlab/psite_annotation). Data processing and visualization were performed using Python (v3.10) using the Pandas, NumPy, and Seaborn libraries. The datasets were filtered to exclude reverse hits and contaminants. Reporter intensities were log10-transformed, and proteins or peptides not quantified across all replicates of at least 1 cell line group were removed. Normalization followed a two-step procedure: first, within-cell line median centering (per batch), and second, bridge-channel-based scaling to correct for batch effects between sample sets (four batches). Missing values were addressed using Perseus-style imputation, drawing from a normal distribution shifted downward by 1.8 σ with a width of 0.3 σ.

#### Kinase Activity Score Calculation

Kinase activity scores were derived by integrating four distinct data matrices, following a modified version of previously established methodologies (40, 43). Kinase abundance from the full proteome was mapped to the UniProt kinase reference (pkinfam, 08.11.2021) and log_10_ transformed. Kinase phosphorylation levels were determined by summing the raw intensities of all associated phosphosites followed by log_10_ transformation. Activation loop phosphorylation was specifically mapped using active loop definitions from Phomics (44), and substrate phosphorylation was quantified by summing the intensities of all known substrates for a given kinase. For each matrix, z-scores were calculated across the experimental conditions using the som R package. A stringent inclusion criterion was applied requiring the presence of kinase abundance data from the full proteome; kinases lacking this information were excluded from the activity score calculation.

#### Statistical Tests

Differential expression analysis for the full proteome and phosphoproteome was performed using two-sample independent Welch’s t-tests implemented via the scipy.stats library in Python (v3.10). Comparisons were conducted between resistant cell lines (BTZ and CFZ) and their respective wild-type (WT) controls across all 4 cell lines. To account for multiple hypothesis testing, *p*-values were adjusted using the Benjamini-Hochberg (FDR) procedure within the statsmodels package. Proteins and phosphosites were considered significantly regulated if they met a dual threshold of an adjusted *p*-value (*q*) < 0.05 and a fold-change ≥2 (log10 ≥ 0.3). Significantly altered kinases (from kinase scores) were identified using a Student's *t* test with Benjamini-Hochberg FDR correction (*q* < 0.05) with an absolute kinase activity score difference greater than 1. Visualizations, including volcano and horizontal bar plots, were generated using ggplot2 and Seaborn.

#### 2D Annotation Enrichment Analysis

To identify co-regulated functional categories between Bortezomib and Carfilzomib resistance, 2D annotation enrichment analysis was performed using Perseus (v1.6.14.0) following the methodology described previously (45). The analysis utilized significant protein lists derived from the BTZ vs. WT and CFZ vs. WT comparisons. Enrichment scores were calculated for functional annotations, and the resulting terms were filtered to include only those with a size ≤500 and a Benjamini-Hochberg false discovery rate (FDR) ≤ 0.01. Visualization of the enrichment results was conducted in R using the ggplot2, ggrepel, and data.table libraries.

## Results and Discussion

### Deep Proteome and Phosphoproteome Profiling of Proteasome Inhibitor-Resistant Multiple Myeloma Cell Lines

**Fig. 1:**
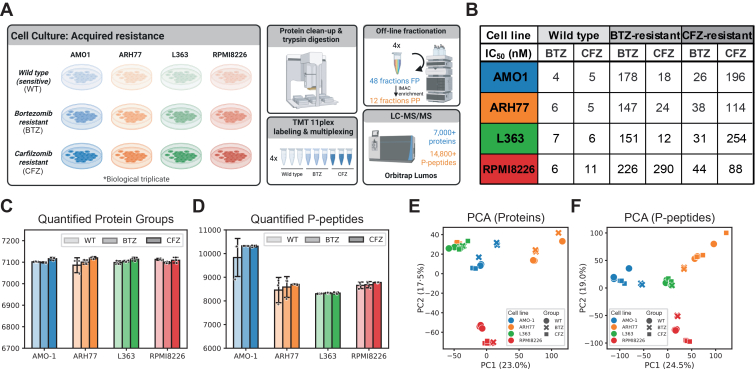
**Proteome and phosphoproteome profiling of proteasome inhibitor-susceptible and resistant multiple myeloma cell lines.***A*, schematic representation of the experimental workflow. Parental (wild-type, WT) and matched bortezomib (BTZ)- and carfilzomib (CFZ)-resistant multiple myeloma cell lines (AMO-1, ARH77, L363, RPMI8226) were cultured in biological triplicates, digested with trypsin, and labelled with TMT 11-plex reagents. Samples were subjected to basic reversed-phase (bRP) fractionation and IMAC phosphopeptide enrichment prior to LC-MS/MS analysis. *B*, table summarizing the half-maximal inhibitory concentrations (IC50, nM) of BTZ and CFZ across all WT and resistant cell lines. *C and D*, Bar plots displaying the total number of quantified proteins (*C*) and phosphopeptides (*D*) across the respective cell lines from three biological replicates. Error bars represent standard deviation (SD). *E and F*, principal component analysis (PCA) of the global proteome (*E*) and phosphoproteome (*F*) datasets.

To investigate the molecular responses associated with proteasome inhibitor (PI) resistance, we performed proteome and phosphoproteome profiling across four diverse myeloma (MM) cell lines using a tandem mass tag (TMT)-based quantitative mass spectrometry workflow (Fig. 1*A*). Bortezomib (BTZ)- and carfilzomib (CFZ)-resistant lines were established from AMO-1 and RPMI8226 (plasmacytoma origin) as well as ARH77 and L363 (plasma cell leukemia origin). Resistance was induced by prolonged exposure to BTZ or CFZ. All resistant lines displayed markedly elevated half-maximal inhibitory concentrations (IC50) compared to their wild-type (WT) counterparts (Fig. 1*B*; Supplementary Fig. S1, *A* and *B*; Supplementary Table S1). Notably, the PI-resistant RPMI8226 lines also exhibited minor cross-resistance to alternate PIs, suggesting a shared mechanism in this cell line.

For proteome and phosphoproteome analysis, cell lysates from WT, BTZ-resistant, and CFZ-resistant variants of each line were prepared in biological triplicates (derived from the same clone, but from different passages). Tryptic peptides were labelled with TMT 11-plex reagents, allowing simultaneous quantification across all conditions in a single cell line. Specifically, each TMT batch included triplicates of the WT, BTZ-resistant, and CFZ-resistant cells from the same parental background. Peptides were fractionated by off-line basic reversed-phase high-performance liquid chromatography (bRP-HPLC) into 48 fractions for full proteome analysis and further pooled into 12 fractions for phosphopeptide (P-peptide) enrichment via IMAC, followed by LC-MS/MS analysis (Fig. 1*A*).

This workflow led to the relative quantification of >7000 protein groups and 8 to 10,000 phosphopeptides per cell line (Fig. 1, *C* and *D*; Supplementary Table S2 and S3). This represents substantially expanded coverage compared to published data from single-line studies such as AMO-1 (19). The biological replicates showed high repeatability, illustrated by tight clustering of cell lines and conditions in principal component analysis (PCA), both for the proteome (Fig. 1*E*) and phosphoproteome (Fig. 1*F*). The dominant factor of variance stemmed from differences between cell lines, consistent with their distinct origins and adaptation trajectories. One notable exception was observed: the CFZ-resistant ARH77 line unexpectedly clustered more closely with the L363 lineage than with its own WT or BTZ-resistant counterparts. This profound phenotypic shift suggests that, under specific selection pressures, myeloma cells may undergo lineage-independent convergence, adopting shared proteomic networks to survive prolonged PI exposure.

### Phosphoproteome Changes in Resistant Lines are Cell Line-Specific and less Pronounced Than Global Proteome Alterations

**Fig. 2:**
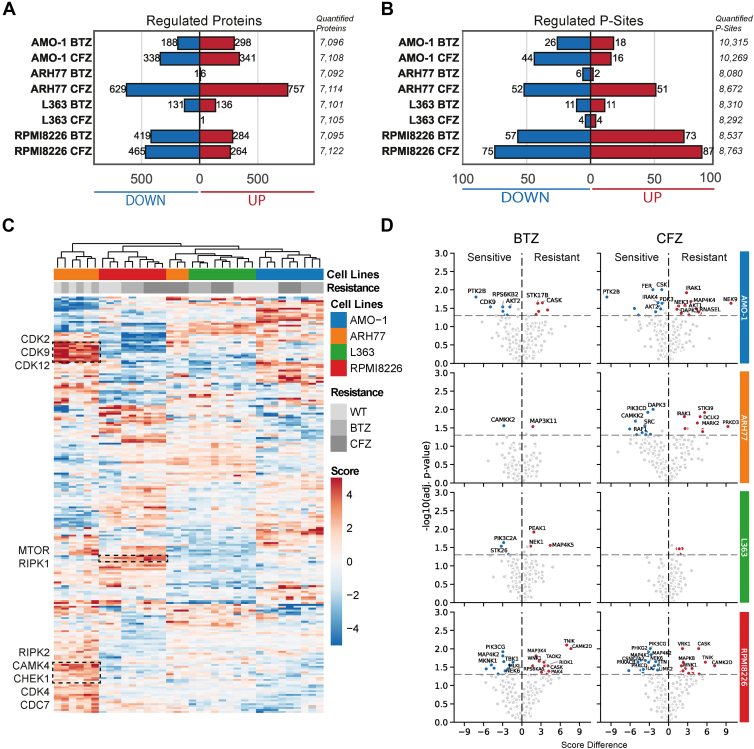
**Global proteome and phosphoproteome changes and kinase activity landscapes in PI-resistant myeloma cells.***A*, Bar plot detailing the number of significantly up- and down-regulated proteins in BTZ- and CFZ-resistant lines compared to their respective WT counterparts. *B*, corresponding bar plot showing the number of significantly regulated phosphosites (P-Sites). For both (*A*) and (*B*), significance was defined as an adjusted *p*-value <0.05 (Welch’s *t* test with Benjamini-Hochberg FDR correction) and a fold-change ≥2. *C*, unsupervised hierarchical clustering heatmap of inferred kinase activity scores across all conditions. *D*, Volcano plots illustrating the changes in kinase activity scores between resistant and WT cells. Kinases were identified as having significantly altered activity if the comparison yielded an adjusted *p*-value <0.05 (Student's *t* test with Benjamini-Hochberg FDR correction) and an absolute score difference >1.

To explore the molecular alterations occurring within the global proteome and phosphoproteome associated with PI resistance, we compared the PI-resistant lines to their respective WT counterparts. We observed ∼260 to 1380 significantly regulated proteins per cell line. Two notable exceptions were the ARH77 BTZ-resistant and L363 CFZ-resistant lines, which exhibited proteome profiles remarkably similar to their WT counterparts (Fig. 2*A*; Supplementary Fig. S2; Supplementary Table S4). Interestingly, the L363 CFZ-resistant line featured only a single up-regulated protein: ABCB1. This well-characterized efflux pump was expressed at levels over 100-fold higher than in the WT line, a known adaptation that confers resistance by actively extruding carfilzomib from the intracellular space (20, 46).

While dynamic phosphoproteome rewiring drives various drug resistance mechanisms in many solid tumors (47, 48), PI-resistant myeloma lines exhibited surprisingly few changes at the phosphorylation level. Excluding the aforementioned outlier lines, an average of only 60 to 162 sites were significantly regulated, indicating that phosphorylation changes associated with resistance adaptations are less prominent and perhaps less permanent than those observed in the global proteome (Fig. 2*B*; Supplementary Fig. S3; Supplementary Table S5).

We investigated this observation in more detail by extracting systems-level information regarding differences in kinase activity in the adaptive state of resistant cells. This was accomplished by computing a kinase activity score based on four metrics (1): kinase abundance (2), kinase phosphorylation (3), kinase activation loop phosphorylation, and (4) substrate phosphorylation, as previously described (40, 43). In total, activity scores for 222 kinases could be computed in this way, providing a fairly comprehensive activity landscape across cell lines (Fig. 2*C*; Supplementary Table S6). Unsupervised hierarchical clustering revealed that activity landscapes were predominantly dictated by the cell line of origin rather than the acquired resistance phenotype, with the exception of the ARH77 CFZ-resistant cells. Specific kinase pathways were activated in different cell-line lineages, such as CDKs and CHEK1 in ARH77, and mTOR in RPMI8226. Similarly, comparing kinase activity scores across resistant and sensitive (WT) cells revealed only slight changes, which would diminish further when lowering the q-value from 5% to 1% (Fig. 2*D*; Supplementary Table S7). While 131 kinases crossed the empirical activity score threshold (>1) and we detected statistically significant abundance changes in 537 phosphorylation sites, these changes should not be overinterpreted. Because multiple myeloma cells exhibit naturally low baseline phosphorylation, the depth of quantifiable substrates is inherently limited. Consequently, our integrated kinase activity metric is dominated by changes in kinase protein abundance rather than specific activity. A kinase may, therefore, cross the chosen significance threshold merely due to protein-level upregulation, without necessitating hyperphosphorylation of its downstream targets. This reinforces the notion that the adaptations driving acquired PI resistance were primarily driven by changes in protein expression.

Interestingly, a recent multi-omic analysis of primary MM patient samples reported that the baseline myeloma (phospho)proteome is already highly deregulated compared to healthy plasma cells as a result of a complex interplay of chromosomal alterations and post-transcriptional regulation (32). Because myeloma cells operate under extreme stress just to manage their high baseline protein production, they may lack the ability to adapt to the acute threat of proteasome inhibition via kinase activity changes or rewiring of signaling. Instead, they appear to be forced into specific and global protein abundance changes (such as the drastic induction of efflux pumps or metabolic enzymes) to overcome drug-induced proteotoxicity. This suggests that effectively targeting PI-refractory disease may require interventions aimed at these global proteomic mediators rather than blocking kinase activity only. This is supported by clinical trial results that showed efficacy when combining kinase inhibition with classical modalities such as PIs, Dexamethasone or Lenalidomide (49).

### Proteome Profiling Reveals NQO1 and NQO2 Upregulation Across Multiple PI-Resistant Cell Lines

**Fig. 3:**
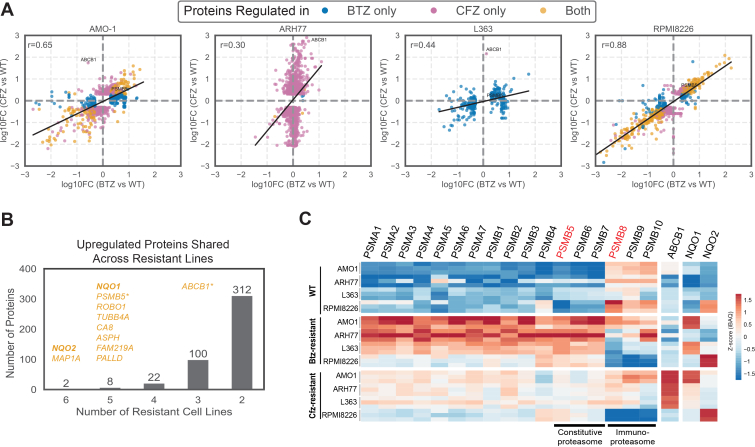
**Identification of protein signatures of PI resistance in MM cell lines.***A*, scatter plots correlating the log10 fold-change of significantly regulated proteins (adjusted *p* < 0.05, fold-change ≥2) in CFZ vs. WT against BTZ vs. WT for AMO-1, ARH77, L363, and RPMI8226 cell lines. Pearson correlation coefficients (*r*) indicate the degree of shared adaptive responses within each lineage. *B*, frequency distribution of consistently upregulated proteins across the eight PI-resistant cell lines. *C*, Heatmap depicting the relative protein abundance (Z-score) of all quantified proteasome subunits, the multidrug efflux pump ABCB1, and the antioxidant flavoenzymes NQO1 and NQO2 across all cell lines and conditions.

Since the most profound molecular adaptations were observed at the proteome level, we next investigated whether any shared resistance mechanisms could be identified from the data. Correlation analysis of protein abundance fold-changes of BTZ vs. WT and CFZ vs. WT for all significantly regulated proteins revealed a highly concordant behaviour in the RPMI8226 resistant lines (*r* = 0.88; Fig. 3*A*), suggesting a broadly similar adaptive response to both PIs. In contrast, the other cell lines displayed moderate (AMO-1, *r* = 0.65) to low correlation (ARH77, *r* = 0.30; L363, *r* = 0.44). Because the ARH77 BTZ-resistant and L363 CFZ-resistant lines exhibited proteome profiles similar to their WT counterparts, the low correlations in these cell lines were driven by the lack of broadly altered protein levels in those cell lines.

To investigate the involvement of broader pathways and biological processes associated with the proteomic shifts upon PI resistance development, we performed 2D annotation enrichment analysis (Supplementary Fig. S4*A*). This approach evaluates pathway regulation simultaneously across two distinct resistance conditions, allowing us to pinpoint core functional adaptations. The analysis clearly identified “Proteasome” as a significantly upregulated functional term in AMO-1, ARH77, and L363 (see also below). Conversely, pathways related to “translation” were consistently downregulated in BTZ-resistant lines but exhibited inconsistent regulation in CFZ-resistant cell lines. The RPMI8226 line emerged as an outlier compared to the other 3 cell lines; while still utilizing similar core resistance mechanisms, it specifically displayed highly upregulated “transcription elongation” and downregulated “immune system” processes (Supplementary Table S8).

To identify potential shared resistance mechanisms driven by single proteins and that might serve as actionable targets in PI-refractory diseases in the future, we filtered for proteins consistently upregulated across multiple resistant cell lines (Fig. 3*B*). We observed that only two proteins, NQO2 and MAP1A, were upregulated in six out of the eight resistant lines. These proteins were detected in all cell lines, but drug response did not reach statistical significance in 2 cell lines. MAP1A (Microtubule-associated protein 1A) is essential for microtubule assembly in cytoskeletal dynamics. While cytoskeletal reorganization is a known facet of cellular stress responses (50, 51), the precise functional contribution of MAP1A to PI resistance in myeloma remains to be elucidated. The other protein, NQO2 (N-ribosyldihydronicotinamide:quinone oxidoreductase 2), is a cytosolic flavoprotein structurally and functionally related to NQO1 (upregulated in five cell lines). It is classically thought to function as a detoxification protein but also plays a critical role in cellular redox regulation. Given that PI-adapted myeloma cells heavily rely on enhanced redox buffering to survive chronic proteotoxic stress (20), NQO1 and NQO2 upregulation may provide an antioxidant shield. Both enzymes also act as gatekeepers of protein degradation for certain proteins such as p53 and have also been shown to reduce proteasome activity by an allosteric mechanism (52, 53). The data also confirmed previously characterized resistance mechanisms, such as the overexpression of the efflux pump and changes in abundance of proteasomal subunits (Fig. 3*C*). For the latter, a broad upregulation is evident, especially in BTZ-resistant cell lines. In contrast, the multidrug transporter ABCB1 is highly upregulated specifically in CFZ-resistant models (with the exception of RPMI8226), confirming its role as a selective CFZ efflux pump. Finally, while both NQO enzymes were frequently upregulated, their basal and drug-induced abundances show lineage-specific differences: NQO1 expression was much higher than NQO2 expression in AMO-1, whereas the opposite was observed in RPMI8226 (Fig. 3*C*; Supplementary Fig. S4*B*).

### Inhibition of NQO2 Re-sensitizes AMO-1 Cells to Carfilzomib

**Fig. 4:**
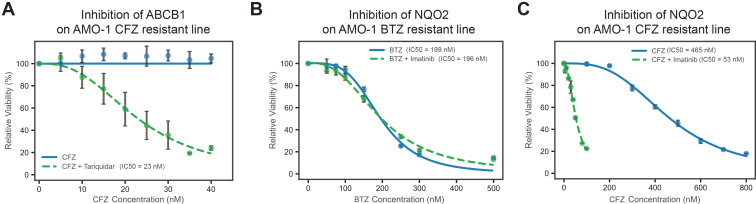
**Inhibition of NQO2 and ABCB1 re-sensitizes CFZ-resistant AMO-1 cells.***A*, viability curves of AMO-1 CFZ-resistant cells treated with low-dose CFZ alone or combined with the ABCB1 inhibitor tariquidar (50 nM). *B*, dose–response viability curves of AMO-1 BTZ-resistant cells treated for 72 h with BTZ alone or in combination with the NQO2 inhibitor imatinib (10 μM). *C*, dose-response viability curves of AMO-1 CFZ-resistant cells treated with CFZ alone or in combination with imatinib (10 μM). Error bars represent the standard error of the mean (SEM) from biological triplicates.

Given the consistent upregulation of NQO2 across cell models, we hypothesized that the protein may contribute to PI resistance. To test this, we selected the PI-resistant AMO-1 models. We first used the ABCB1 inhibitor tariquidar (50 nM) as a positive control to show that it can break CFZ resistance caused by ABCB1 overexpression (Fig. 4*A*). For NQO2, we chose imatinib, a clinically approved kinase inhibitor for which NQO2 is a very potent off-target. Resistant cells were treated for 72 h with PIs (dose-dependent BTZ or CFZ) either as single agents or in combination with a high dose of imatinib (10 μM) to ensure full inhibition of enzymatic activity. In the AMO-1 BTZ-resistant line, single-agent PI treatment maintained the expected resistance phenotype and co-treatment with imatinib had no effect (Fig. 4*B*; Supplementary Table S9).

In contrast, imatinib, which does not inhibit NQO1, collapsed resistance to carfilzomib (Fig. 4*C*). The proteome profile indicated that NQO2 basal expression is relatively low compared to NQO1 in AMO-1 WT cells (Supplementary Fig. S4*B*). The drastic drug phenotype underscores that protein abundance alone does not necessarily equate to functional essentiality as even at low basal levels, NQO2 activity appeared to be a critical contributor to CFZ-resistance. As the reported IC50 of imatinib for NQO2 in recombinant enzyme assays is ∼40 nM (54), the high dose (10 μM) of imatinib was likely sufficient to inhibit 100% of NQO2 activity in cells. Even though the exact mechanism of how NQO2 inhibition leads to restoring CFZ sensitivity requires further research, these results highlight a theoretically therapeutically actionable strategy in CFZ-resistant MM by combining CZF with imatinib (with and without blockade of ABCB1 by tariquidar) because NQO2 activity will be fully blocked at the standard clinical dosing of imatinib. Such a combination may be acceptable because NQO2 inhibition is generally not toxic to cells. A similar approach may not be possible for MAP1A, the second protein most consistently upregulated in CFZ-resistant AMO-1 cells, because targeting microtubules by molecules such as maytansine will likely lead to unacceptable toxicity. However, further translational studies are required to substantiate these projections. Lineage-specific reliance on different NQO paralogs (Fig. 3*C*) may also require paralog-specific stratification or pan-NQO inhibition to achieve maximal effects. Ongoing work in the laboratories of the authors extends these investigations to further cell lines, proteins and inhibitors. Regardless, the data generated in this study may serve as a valuable molecular resource to the community and starting point for discovering additional proteasome resistance mechanisms in multiple myeloma.

## Data Availability

The mass spectrometry proteomics data have been deposited to the ProteomeXchange Consortium via the PRIDE (55) partner repository with the dataset identifier PXD077254.

## Supplemental data

This article contains supplemental data.

## Conflict of Interest

The authors declare that they have no conflicts of interest with the contents of this article.
