# Supplementary material for: Proteome Profiling Reveals NQO2 Activity Contributing to Proteasome Inhibitor Resistance in Multiple Myeloma Cell Lines
Source: Mol Cell Proteomics. 2026 Jun 26;25(8):101615. doi: 10.1016/j.mcpro.2026.101615 (PMC13396865; doi:10.1016/j.mcpro.2026.101615)
Supplement: Supplemental Figures [file mmc10.docx]

Supplementary Figures


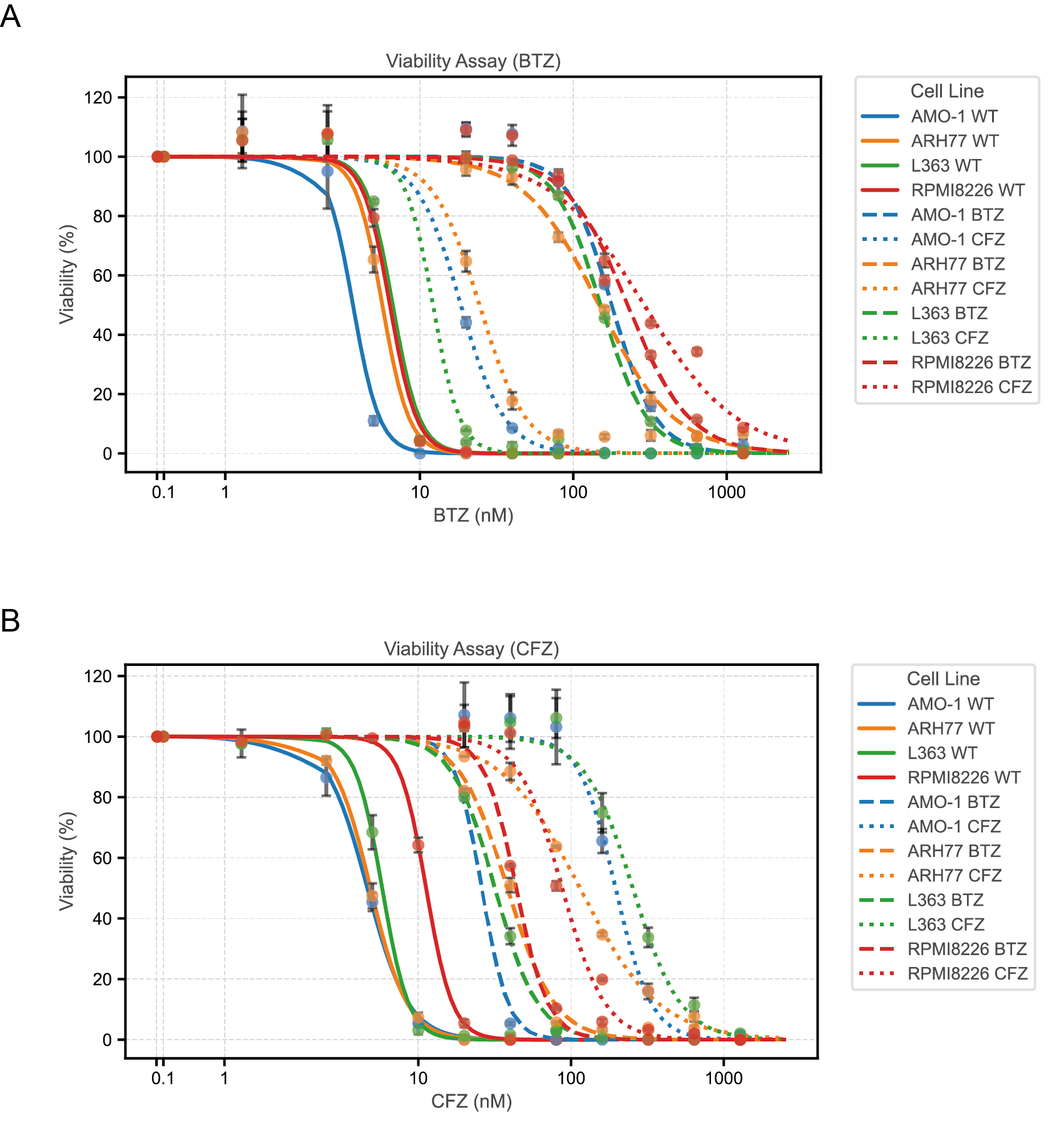


**Supplementary Figure S1. Validation of acquired proteasome inhibitor resistance in multiple myeloma cell line models.**

Dose-response viability curves of wild-type (WT) and paired bortezomib (BTZ)- and carfilzomib (CFZ)-resistant cell lines (AMO-1, ARH77, L363, and RPMI8226). Cells were treated with varying concentrations of BTZ (A) or CFZ (B) for 72 hours, and viability was assessed using the Alamar Blue assay. Error bars represent the standard error of the mean (SEM) from biological triplicates.


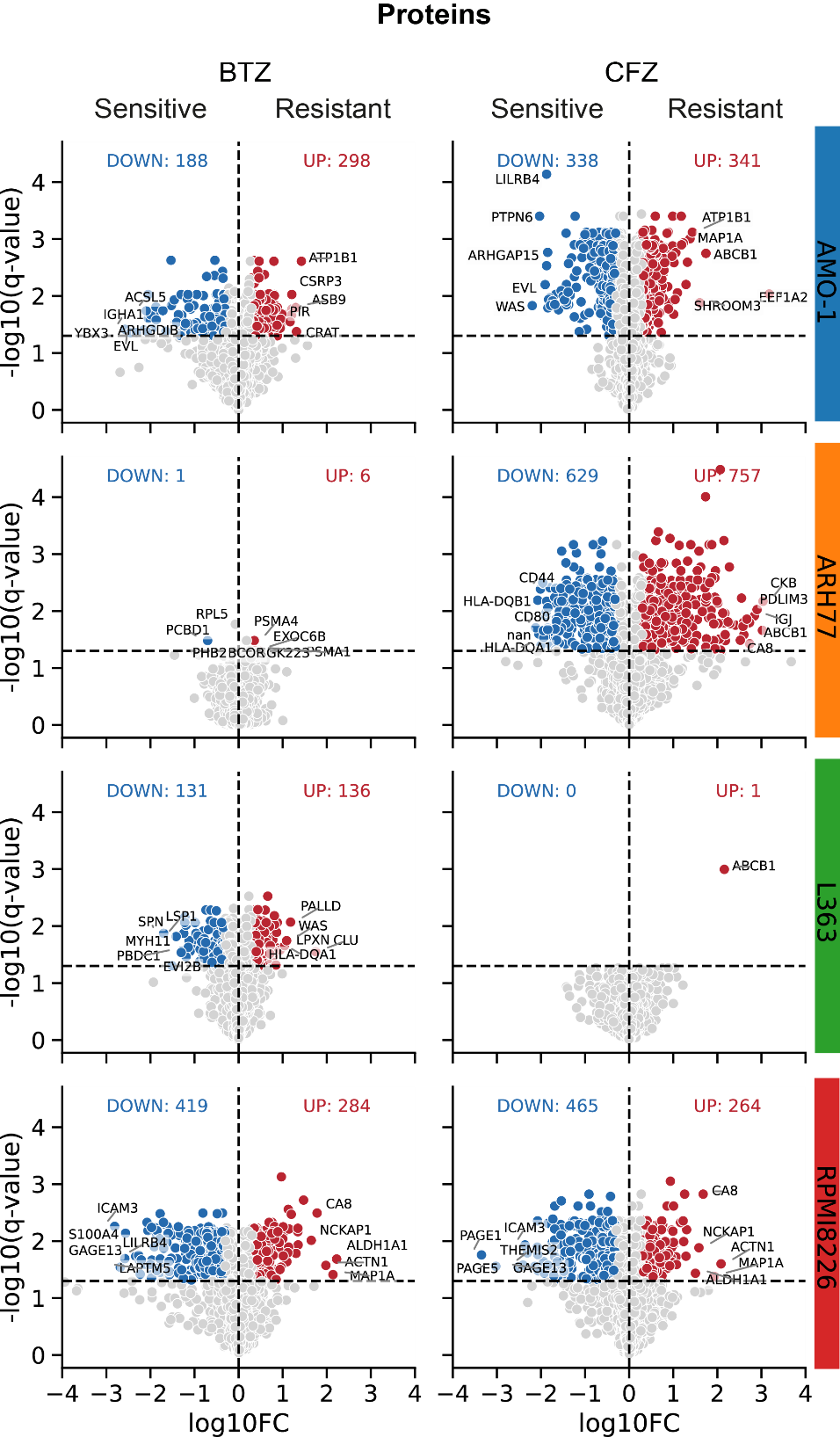


**Supplementary Figure S2. Global proteome changes across PI-resistant multiple myeloma cell lines.**

Volcano plots displaying the global log_10_ fold-change versus −log_10_(adjusted *p*-value) for quantified proteins in bortezomib (BTZ)- and carfilzomib (CFZ)-resistant lines compared to their respective wild-type (WT) controls across the AMO-1, ARH77, L363, and RPMI8226 lineages. Significantly up- and downregulated proteins are highlighted. Statistical significance thresholds (indicated by dashed lines) were defined as an adjusted *p*-value < 0.05 (two-sample independent Welch’s t-test with Benjamini-Hochberg FDR correction) and a fold-change ≥ 2.


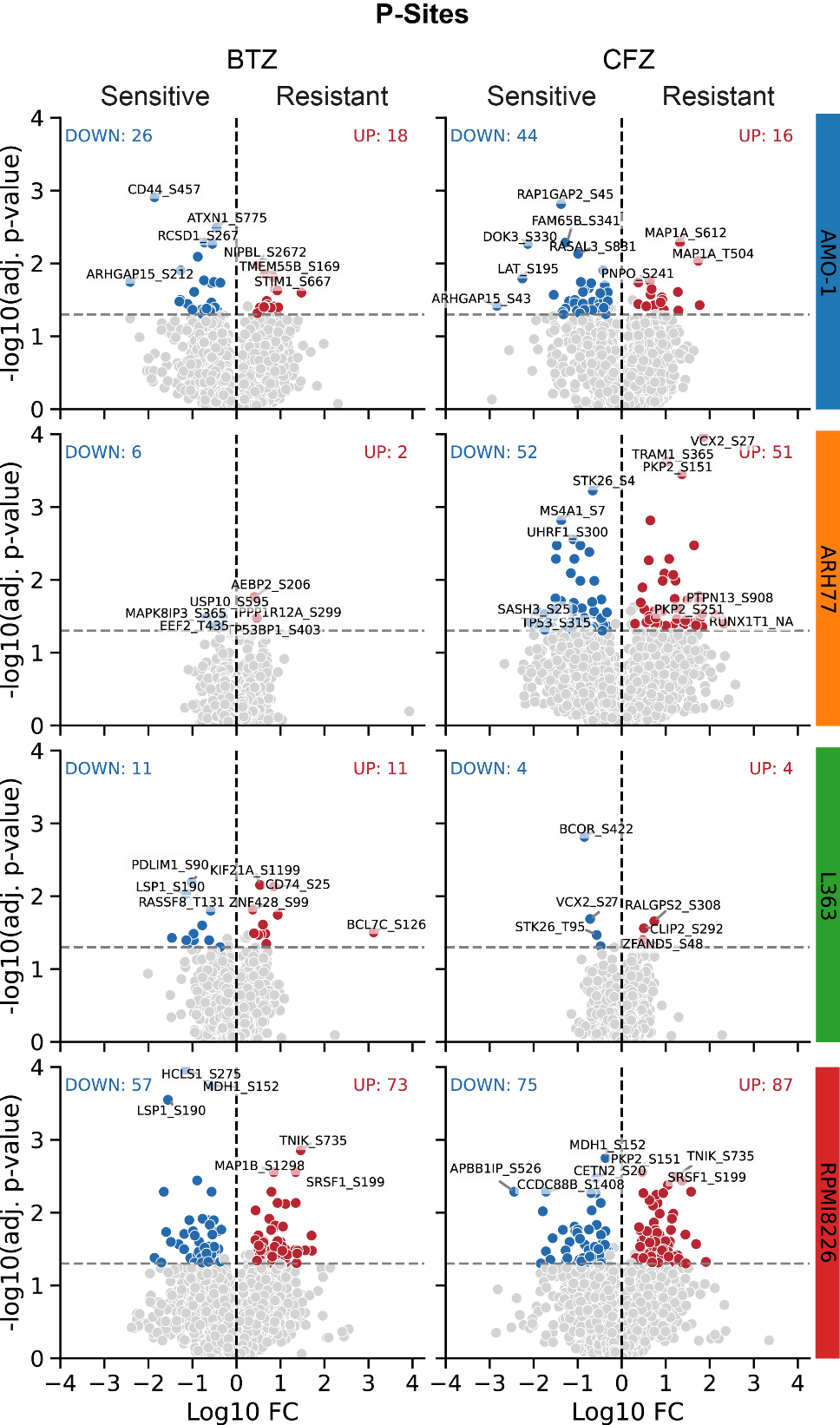


**Supplementary Figure S3. Global phosphoproteome changes across PI-resistant multiple myeloma cell lines.**

Volcano plots detailing differentially regulated phosphosites in BTZ- and CFZ-resistant lines compared to their respective WT controls across the four myeloma lineages. The axes and statistical significance thresholds (adjusted *p*-value < 0.05, fold-change ≥ 2) are identical to those applied to the global proteome in Supplementary Figure S2.


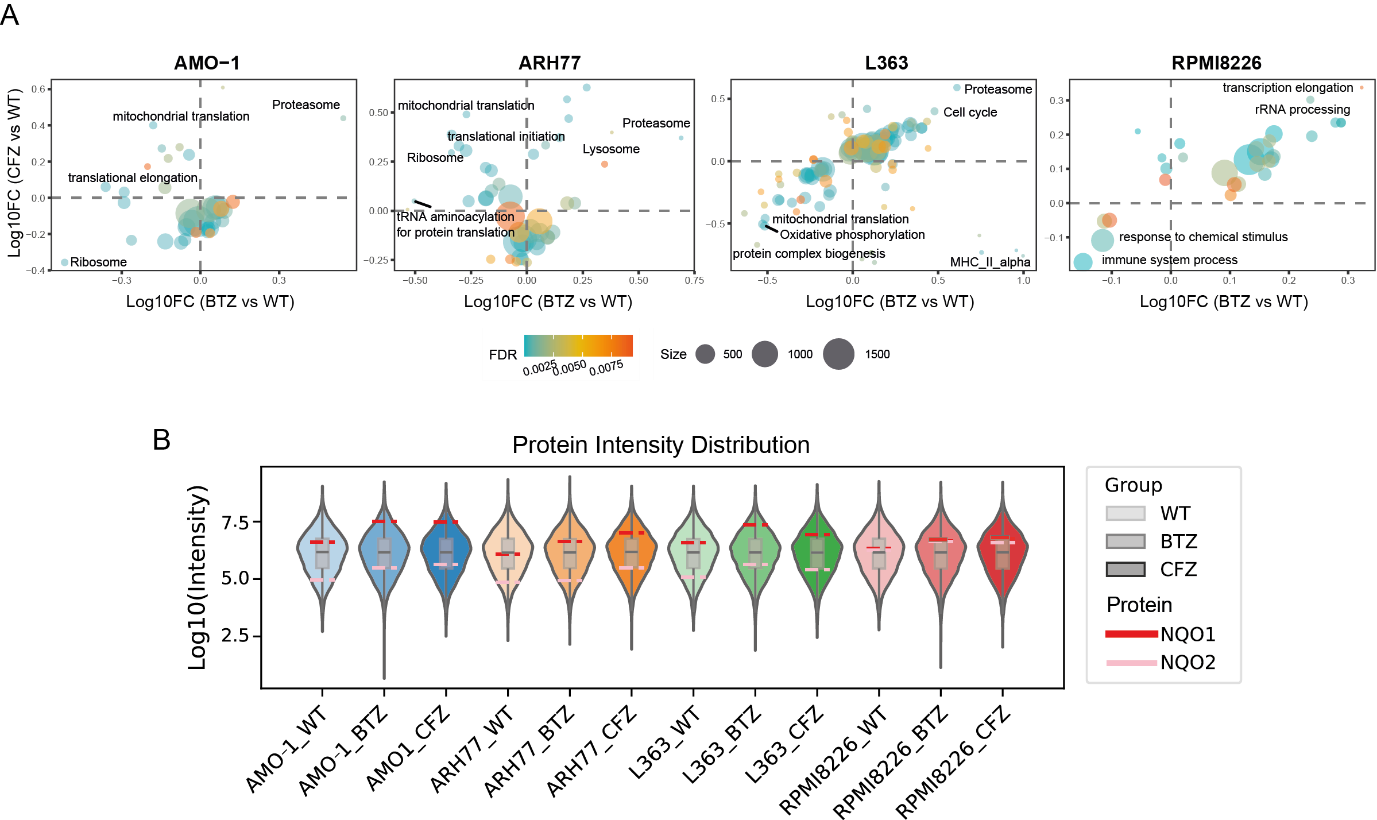


**Supplementary Figure S4. 2D annotation enrichment analysis and cellular abundance of NQO1/2 across PI-resistant multiple myeloma cell lines.**

(A) 2D annotation enrichment scatter plots highlighting co-regulated functional categories between bortezomib (BTZ) and carfilzomib (CFZ) resistance. Analyses were performed using Perseus, comparing significant protein lists from BTZ vs. WT and CFZ vs. WT. Each data point represents a functional annotation term (filtered for a category size ≤ 500), plotted by its respective enrichment scores. Node size corresponds to the number of proteins associated with the term, and the color gradient indicates statistical significance (Benjamini-Hochberg FDR ≤ 0.01). (B) Violin plots showing the relative cellular protein abundance of the antioxidant flavoenzymes NQO1 and NQO2 across the WT, BTZ-resistant, and CFZ-resistant lineages.
